# Acknowledgement to Reviewers of *Veterinary Sciences* in 2014

**DOI:** 10.3390/vetsci2010012

**Published:** 2015-01-09

**Authors:** Veterinary Sciences Editorial Office

**Affiliations:** MDPI AG, Klybeckstrasse 64, CH-4057 Basel, Switzerland

The editors of *Veterinary Sciences* would like to express their sincere gratitude to the following reviewers for assessing manuscripts in 2014:

Ahmed, Ahmed
Alves, Frauke
Alvito, Paula Cristina
Bach, Jean-François
Belkoff, Stephen M.
Bennett, Peter
Bjørnvad, Charlotte Reinhard
Bloom, Paul
Brown, Mark A.
Canonica, Giorgio Walter
Doghman, Mabrouka
Foley, Janet
Gallo-Payet, Nicole
Gilch, Sabine
Gordon, Bruce R.
Haanstra, Krista
Irwin, Peter J.
Kang, Seung Won
Langdon, Simon P.
Langeveld, Jan
Liu, Fu-Tong
Matsumoto, Taro
Mestre-Francés, Nadine
Meyer, Hermann
Norman, Elizabeth J.
Plesker, Roland
Rasgon, Jason L
Raverty, Stephen
Reber, Stefan
Richards, Kristy L.
Rüfenacht, Daniel
Salzer, Johanna
Sbiera, Silviu
Schmidt, Mathias
Suchecki, Deborah
Tanaka, Tetsuya
Telford, Sam
Val, Pierre
Vialou, Vincent
Vitale, Maria
Williams, Bart O.

